# Next-Generation Digital Ecosystem for Climate Data Mining and Knowledge Discovery: A Review of Digital Data Collection Technologies

**DOI:** 10.3389/fdata.2020.00029

**Published:** 2020-09-10

**Authors:** Angel Hsu, Willie Khoo, Nihit Goyal, Martin Wainstein

**Affiliations:** ^1^Yale-NUS College, Singapore, Singapore; ^2^Department of Public Policy, University of North Carolina-Chapel Hill, Chapel Hill, NC, United States; ^3^Yale OpenLab, New Haven, CT, United States

**Keywords:** climate data, earth observation, internet of things, blockchain technology, climate policy, public policy, climate change mitigation, big data

## Abstract

Climate change has been called “the defining challenge of our age” and yet the global community lacks adequate information to understand whether actions to address it are succeeding or failing to mitigate it. The emergence of technologies such as earth observation (EO) and Internet-of-Things (IoT) promises to provide new advances in data collection for monitoring climate change mitigation, particularly where traditional means of data exploration and analysis, such as government-led statistical census efforts, are costly and time consuming. In this review article, we examine the extent to which digital data technologies, such as EO (e.g., remote sensing satellites, unmanned aerial vehicles or UAVs, generally from space) and IoT (e.g., smart meters, sensors, and actuators, generally from the ground) can address existing gaps that impede efforts to evaluate progress toward global climate change mitigation. We argue that there is underexplored potential for EO and IoT to advance large-scale data generation that can be translated to improve climate change data collection. Finally, we discuss how a system employing digital data collection technologies could leverage advances in distributed ledger technologies to address concerns of transparency, privacy, and data governance.

## Introduction

The advent of big data analytics and development of earth observation (EO) and internet of things (IoT) technologies have, over the preceding decade, opened up new fields of research and practice, ones that center on database mining and knowledge discovery. In the context of EO, methodologies of spatial data mining and knowledge discovery aim to extract and analyze large, high-dimensional, and complex information (Mennis and Guo, 2009). In the context of IoT, Tsai et al. (2014b) argue that a future digital ecosystem will not only involve making “things” smart and scaling up the internet infrastructure to handle the exponential growth in connected devices, but also integrating data mining to provide better services through new functionalities such as association analysis, classification, clustering, outlier analysis, and time-series analysis (Chen et al., 2015).

A growing area of scholarship proposes ideas for constructing an intelligent digital ecosystem that can harness this big data potential (Marjani et al., 2017). Researchers have introduced new brands of IoT, such as “Future Internet of Things” (Tsai et al., 2014a), “Cognitive Internet of Things” (Wu et al., 2014), and a “Social Internet of Things” (Gil et al., 2016). While these proposals envision differing relationships between data and analysis, they all agree on the need to combine IoT with analytics to translate data into knowledge. Integrating IoT, analytics, and cloud computing would enable what Chen et al. (2018) have called “cognitive computing.” The specific paradigm and system architecture for combining data collection technologies, analytics, and data storage and retrieval will largely depend on the digital services required of the field of research in question. For the emerging sphere of “big Earth data” or “big environmental data” that speaks to the increasing volume, variety, and velocity with which technological advances in EO and IoT are generating massive amounts of data relevant for urgent issues like climate change, there is both great promise and challenges with harnessing this potential and translating it to usable insight (Sudmanns et al., 2018).

Climate change has been dubbed “the defining challenge of our age” (Rosenthal, 2007). Yet scientists and policymakers continue to lack adequate data to understand how we are progressing in terms of mitigating impacts. With global temperatures already 1.1 degrees C above pre-industrial levels [World Meteorological Organization (WMO), 2019] and dangerously close to thresholds where scientists agree climate change's effects will be irreversible (IPCC, 2014a, 2018; Jackson et al., 2019), the need to address data gaps with respect to climate change could not be more urgent. Although data and models to understand anthropogenic climate change have greatly advanced since the first reports establishing the scientific basis of climate change (IPCC, 1990), uncertainties still exist (Soden et al., 2018). Estimates of anthropogenic-induced emissions and socioeconomic drivers are primarily derived from climate models, which rely on global earth observation data derived from ground-based and satellite measurements (Guo et al., 2015). The relatively coarse spatial resolution (~100 sq km) of a typical climate model (IPCC, 2007) is generally too crude to evaluate local impacts or climate change actions at the individual actor level (Hsu et al., 2019). Particularly in the policy domain, more fine-grained data are needed to evaluate policy and program performance and to understand whether actions to mitigate greenhouse gas emissions or adapt to climate change impacts are being achieved.

The emergence of digital data collection technologies, such as satellite remote sensing and low-cost sensors, promises to provide new advances in data collection and monitoring for the Sustainable Development Goals (SDGs), particularly where traditional means of data collection, such as government-led statistical census efforts, are costly and time consuming (Fritz et al., 2019; Anenberg et al., 2020). Despite these technological advances, their uptake into policy processes and applications has been relatively limited (Fritz et al., 2019). Recent research efforts, however, have been initiated to better connect the digital technology, computer science, and data science communities with climate change science, policy and practitioner communities, including the Climate Change AI effort (Rolnick et al., 2019). In this initiative, a coalition of industry and academic experts identify 13 sectors related to climate change, including buildings, transport, industry, and agriculture, where advances in machine learning (ML) can be applied to empower work in climate change (Rolnick et al., 2019). While the authors touch upon ML methods applications for collective decision-making and policy, more elaboration is needed to bridge cross-sector digital tech-enabled data collection and monitoring to the question of climate policy and climate action tracking.

This article examines the extent to which digital data technologies, such as EO technologies (e.g., remote sensing satellites, unmanned aerial vehicles or UAVs, generally from space) and IoT (e.g., smart meters and sensors, generally from the ground) can address existing data gaps that stymy efforts to measure climate change mitigation progress. Although numerous reviews of EO (Navalgund et al., 2007; Pettorelli et al., 2014; De Araujo Barbosa et al., 2015) and IoT (Atzori et al., 2010; Stankovic, 2014; Al-Fuqaha et al., 2015) exist, to the best of our knowledge, this is the first integrated, comprehensive review of the application of such digital data collection technologies in the domain of climate change.

Our aim is that this review motivates a future research agenda to explore the potential for digital data collection technologies to bridge gaps in climate change data collection. While digital data collection technologies alone are not silver bullet solutions, there is underexplored potential for EO and IoT to advance large-scale data generation to assess progress of climate change mitigation efforts. We end our review with a discussion of how a system architecture combining digital data collection technologies could leverage advances in distributed ledger technology (DLT) to integrate these new data mining streams alongside legacy databases in a way that addresses transparency, data validation, and privacy concerns. In evidence-based policy in particular, there is increasing demand for transparency and traceability not just in government, agency, and policymaker actions, but in the documentation of original data and analytic processes used to evaluate them (Sicilia and Visvizi, 2019). DLT-based systems could provide a level of permanent availability, immutability, and tamper-resistant evidence that is now becoming the burden of proof when demonstrating the efficacy of policy actions. With this discussion, we aim to stimulate future research related to DLTs for collecting and coalescing climate data.

## Defining the Problem Space: Issues With Data Collection

Although there is no consensus defining exactly what climate change data constitutes, for the purposes of this review article, we are concerned mainly with data that is a “time series of measurements of sufficient length, consistency and continuity to determine climate variability and change,” often referred to as a Climate Data Record or CDR (National Center for Atmospheric Research Staff, 2014). Exactly how climate variability and change are defined, are also of scientific debate, but in this review we more narrowly consider CDRs in the context of climate-warming greenhouse gas emissions (GHGs) that have “iconic status in climate science as evidence of the effect of human activities on the chemical composition of the global atmosphere” (Le Treut et al., 2007). These anthropogenic GHG-emitting activities mostly involve the combustion of fossil fuels for electricity generation and transport, industrial processes, and land-use change (Stocker et al., 2013). In a policy context, not only does the concentration of GHGs in the atmosphere matter, but also their attribution to specific sources and actors is an important consideration in determining whether actions to mitigate or reduce climate change are effective. Towards that aim, a GHG inventory that combines data from various emissions sources within a defined boundary is considered the foundation for binding policy commitments and performance evaluation (Peters and Hertwich, 2008). GHG inventories can be produced at multiple levels from the individual actor level to national and global scales, and they are critical tools for policymakers to develop mitigation strategies and track the progress of their implementation, as well as for agencies and businesses to comply with regulations or identify opportunities for better management [World Resources Institute (WRI) and World Business Council for Sustainable Development (WBCSD), 2011; Jonas et al., 2019].

Data collection for tracking climate mitigation progress, however, suffers from several overarching challenges. First, GHG inventories are largely limited to self-reported data. Most actors that do report data are disproportionately located in the global North (Hsu et al., 2019), making it difficult to obtain a complete picture of anthropogenic impact on the global climate, particularly given the increasing contribution of global South actors to GHG emissions (Climate Watch, 2019). Second, GHG emissions are rarely measured directly and instead primarily estimated using activity data (i.e., amount of fuel consumed, vehicle miles traveled, etc.), which is inherently problematic because not every activity's impact on climate emissions can be accurately quantified (Jonas et al., 2019). Third, because data are self-reported and calculated from activity data, their accuracy and completeness suffer from what Paterson and Stripple (2010) note as numerical choices, which can serve to render visible some things and visible others. As a result, an emissions inventory never truly provides a complete picture of a company's impact on climate change (Walenta, 2018). Fourth, there is a lack of methodological consistency and comparability between actors' inventories due to decisions in what emission sources to include and what calculation methods to apply. In evaluating climate policies' impact, there are also measurement and data collection uncertainties related to the efficacy of some policy instruments (e.g., emissions trading vs. education campaigns; Markolf et al., 2017; Jonas et al., 2019), but for this review we are more narrowly focusing on challenges related to emissions data inventories, where we see potential for technology-enabled solutions. We organize these data collection issues into several points of discussion below: standardization, resolution, completeness and certainty, transparency, and other technical barriers.

### Lack of Standardization

Individual actors, ranging from companies and organizations to subnational and national governments and even citizens, can self-report emissions data using a suite of calculation methodologies and protocols. The Intergovernmental Panel on Climate Change (IPCC)—the leading scientific body on climate change science—presents several tiers of methodological complexity for calculating national greenhouse gas emissions inventories (IPCC, 2006), but essentially they involve relying on activity data (e.g., fuel combustion in stationary sources) multiplied by emissions factors (e.g., GHG emissions generated per unit activity or fuel type). Based on these methods, non-profit organizations and think-tanks, primarily the World Resources Institute and the World Business Council for Sustainable Development, have adapted a corporate and organization-level accounting method called The Greenhouse Gas Protocol [World Resources Institute (WRI) and World Business Council for Sustainable Development (WBCSD), 2011], which provides more detailed guidance for inventory development for smaller-scale actors and was formally adopted by the International Standards Organization (ISO, 2006). Considerations such as inventory boundary, emission scopes (i.e., direct vs. indirect), and emission sources are under an actor's discretion to report. Due to these methodological choices, resulting emissions inventories between actors are not necessarily comparable.

For local government actors, the IPCC (2014b) identifies a lack of systematic evaluation of urban climate policy plans and their effectiveness, given the lack of consistent and comparable accounting to assess their impact (Zimmerman and Faris, 2011). While there are some efforts in place to standardize emissions inventory calculations for cities and communities, such as the Global Protocol for Community-Scale Greenhouse Gas Emission Inventories (Fong et al., 2014), subnational governments do not necessarily measure and report inventories in consistent ways nor do they transparently communicate what assumptions or areas of uncertainty are associated with reported data. Some efforts, like the Global Covenant of Mayors, a transnational climate initiative that includes nearly 10,000 cities, are working toward a common reporting framework and guidelines for their members, which should help improve standardization [Global Covenant for Mayors Energy (GCOM), 2018]. Current lack of standardization means there exists very little comparable greenhouse gas emissions baseline or inventory data by which to compare actors or develop an aggregate picture of global progress.

### Low Resolution (Temporal and Spatial)

Ideally, data monitoring climate impacts and mitigation performance would be available for multiple years, be spatially relevant (e.g., appropriate spatial resolution for a unit of analysis) and spatially explicit (e.g., for appropriate attribution). Because the process of “mining” activity data to conduct an inventory using existing standards and protocols, which as described above requires collection of fuel or activity data and determination of appropriate emission factors, is time consuming, it is challenging for actors to regularly report annual emissions inventories (Ogle et al., 2013; Markolf et al., 2017). CDP (formerly Carbon Disclosure Project) estimates it takes upwards of 100 h to complete their annual Supply Chain Questionnaire for corporations, and, in 2014, only 3,500 companies disclosed emissions information (Anderson, 2015). For the Compact of States and Regions, a transnational climate initiative that includes 124 actors from 35 countries around the world, their annual disclosure and analysis report has only been able to feature the same 44 actors due to lack of reporting from other actors (The Climate Group, 2015, 2016, 2017). Those that do report emissions inventory data also may not be the most significant actors. In a recent report, it was found that 70 percent of 1,500 of the companies with the largest impact on the world's forests failed to disclose their data and their role in deforestation (CDP, 2019). Particularly for agriculture and land-use based emissions, a critical gap exists in spatially-explicit data on land-use changes that have inherently different impacts on emissions, and are especially poor for developing countries (Smith et al., 2012; Ogle et al., 2013). While researchers have experimented and proposed statistical modeling approaches to develop spatially-explicit, temporal emission inventories, their development is “extremely labor-intensive” and “only available for limited places and times,” with only select coverage in a few locations due to the lack of physical measurements that can be used to ground-truth modeled data (Oda et al., 2019).

### Incomplete and Uncertain Data

Many aspects of climate mitigation suffer from incomplete data for evaluating performance. There are some sectors and categories of emissions that are frequently excluded from emissions inventories that are relevant for actors' mitigation efforts. For example, many cities, and companies as well, do not account for “Scope 3” or indirect emissions resulting from upstream or downstream supply chain or embodied carbon emissions. For many actors, these indirect or Scope 3 emissions could represent significant contributions to their footprint (Ramaswami et al., 2008). For example, the U.S. city of Portland, Oregon, found that 58 percent of their carbon emissions were derived from these Scope 3 emissions sources when they conducted a consumption-based approach to accounting [Portland Bureau of Planning Sustainability (PBPS), 2015]. Kennedy et al. (2009) found that upstream emissions due to the extraction, processing and transporting of fossil fuels could add between 7 and 24 percent to cities' end-use emission totals. Further, land-sector and land-use based emissions in the agriculture and forestry sectors that are critical to assess as both carbon sinks and sources are methodologically challenging to assess due to differences in assumptions related to carbon cycle models, quantification of rates and dynamics of land-cover change, soil responses to land-cover change, baseline carbon stocks in vegetation and soils, among other issues (Ramankutty et al., 2007), which is why their estimation is often missing or fraught with uncertainties.

Where emissions inventories are available, they primarily exist in industrialized countries. The process of developing emissions inventories is time consuming and costly. There is evidence to suggest that the time, resources, and capacity needed to complete an emissions inventory can actually inhibit the implementation of a climate mitigation strategy itself (Markolf et al., 2017). Sparse emissions data exist for developing and emerging countries (Bulkeley et al., 2012; Pattberg et al., 2012; Chan et al., 2018; NewClimate Institute et al., 2019), which will drive emissions and population growth over the next several decades [United Nations Framework Convention on Climate Change (UNFCCC), 2019; US Energy Information Administration (E.I.A.), 2019] and are especially vulnerable to the impacts of climate change (IPCC, 2014a). The resources needed to report and measure climate actions may play a role in the data gaps in the global South, for instance, as well as in small and medium enterprises and less wealthy cities and states within developed economies. Actors in these countries face both human resource capacity challenges (e.g., expertise, lack of clearly designated roles in relevant government agencies for producing inventories, insufficient documentation and archival systems) and technical issues (e.g., incomplete or non-existent activity data or lack of experimental data for developing country or technology-specific emission factors) for producing emissions inventories (Ogle et al., 2013). Developing country fossil-fuel based CO_2_-emissions inventories are estimated to be two to three times less accurate than those in developed countries (Ciais et al., 2015).

Compounding their incompleteness studies evaluating the quality of existing emission inventory data point to their inherent uncertainty. Uncertainty of greenhouse gas emissions data has been estimated to have increased, largely due to uncertainties in carbon uptake by sinks such as oceans and lands (Ballantyne et al., 2012). In terms of bottom-up derived accounting of emissions-generating activity data and modeling, Duren and Miller (2012) estimate uncertainty in the order of 5–20 percent per year for CO_2_ emissions from individual countries. One study found a gigatonne difference between CO_2_ emission inventories for China and its 30 constituent provinces for the time period 1997–2010 based on two publicly available energy data sets (i.e., activity data) by which to estimate emissions (Guan et al., 2012). This amount is roughly equivalent to Japan's annual CO_2_ emissions or five percent of the global total. Even small ranges of uncertainty, when multiplied across space and time, can have significant influence over the accuracy of emissions data and relevance for understanding global progress toward climate goals.

### Lack of Transparency

Transparency is a key challenge that prevents comprehensive tracking and analysis of climate mitigation. Approximately 6,000 of the 10,400 cities and states and 1,500 of the 6,000 companies recording climate action in 2018 shared information that enabled researchers to quantify the potential mitigation impact of these actions (NewClimate Institute et al., 2019). Very few actors, however, share data tracking their progress toward implementing mitigation policies (Hsu et al., 2019; NewClimate Institute et al., 2019), which is why there have been calls for measurement, reporting and verification (MRV) procedures for international climate agreements specifically and accountability for other climate actions that could be perceived as greenwashing (Taebi and Safari, 2017). Concerns about the proprietary and confidential nature of data further prevents free and open exchange of climate-related data relevant for tracking progress.

### Other Technical Barriers

For some sectors, measuring emissions and reductions due to management or policy efforts is a technically challenging undertaking. Determination of emissions savings depends on the type of activity as well as the greenhouse gas targeted (Jonas et al., 2019). Particularly for non-CO_2_ gases, such as methane, which can result from leakages at oil and gas sites or from agricultural processes, emissions are difficult to directly measure or estimate. There is also a lack of agreed-upon methods for assessing land-use and agriculture-sector emissions, which, for many actors, comprise the bulk of their emissions footprints (Forsell et al., 2016). Livestock production and management practices, for example, can vary substantially between countries and introduce a range of uncertainty in emissions from these sources (Ogle et al., 2013). Uncertainty in estimation for these land-based emissions and removals have greater uncertainty than fossil-fuel based emissions, with estimates of nitrous oxide (N_2_O) more uncertain than either methane or CO_2_ (Jonas et al., 2019). In most national inventories, the high spatial and temporal variability of N_2_O emissions dominates overall uncertainty – the underlying measurements and data are so unreliable that governments have to apply the lowest tier of IPCC estimation to include them in their reports (Leip et al., 2011).

Measuring the performance of climate action commitments focused on functions besides mitigation is even more difficult, including progress on adaptation to climate change, which is often defined as the reduction of harm from current and future climate risks as measured through averted impacts (Ford et al., 2015). Efforts to track adaptation face the lack of a consistent definition of adaptation activities, baselines or benchmarks by which to assess progress, the lack of systematic reporting on adaptation progress, and insufficiently large-scale data by which to assess progress (Ford et al., 2015). The varying adaptation challenges and resources different countries, cities, regions, and companies encounter also make a common standard for comparing and measuring different adaptation efforts challenging (Berrang-Ford et al., 2019).

## Potential Digital Data Collection Solutions

Given the data gaps in climate action tracking, new technologies and sources of data could be leveraged to complement existing data collection efforts. This section reviews potential solutions according to the categories of data gaps established in the previous section. We survey developments in Earth Observation (EO) and Internet of Things (IoT) technology, both of which have the potential to capture robust data on climate, natural processes and human activities that can complement or act as proxies to assess progress on climate change mitigation. Table 1 provides a summary of these solutions alongside their limitations. For this paper, we distinguish between EO technology as space-based and near-earth sensors (e.g., satellites, drones) and ground-based IoT technology that primarily comes in the form of sensors and networks (e.g., soil moisture sensors and control centers in precision agriculture) as a way of organizing our review.

**Table 1:** Climate mitigation tracking challenges addressed by EO and IoT technology.

**Challenges addressed**	**EO**	**IoT**
Lack of standardization	Partially + The EO community adheres to internationally shared guiding principles and reference standards for data collection, processing, and dissemination – Measurements between sensors are not always comparable; accuracy of measurements may vary depending on ground-truth data available for validation	No – Plethora of standards and protocols
Low spatiotemporal resolution	Partially + Spectral imaging and radar sensing could provide data at high spatiotemporal resolutions, although attribution to specific actors could still pose a problem – Space-based and near-earth EO-based measurement and GHG inventory development most robust when combined with ground-based measurements, which are limited across the world	Yes + Near-real time/real time and continuous monitoring and transmission of data
Incomplete data	Partially + Technological advances, lowering cost of satellites and UAVs could increase deployment – EO-derived greenhouse gas emission data still largely experimental, would need to be combined with other ground-based or multi-layered data to demonstrate robustness and accuracy – Current generation of CO_2_-monitoring satellites not capable of attributing/pinpointing emission sources.	Yes + Low cost of development and production of devices)
Lack of transparency	Yes + Reduced reliance on climate actors to share or self-report data	Partially + Rise of citizen science using IoT devices could pave the way for new modes of climate tracking – Potential privacy concerns that need to be addressed before IoT scaled widely at the individual level
Other technical barriers	Yes + Significant potential and technological development of remote sensors that can measure non-CO_2_ gases, such as methane, which can result from leakages at industrial or agricultural sites	Yes + Sturdy and versatile devices can collect difficult to obtain emissions data in harsh environmental conditions

### Earth Observation (EO) Data

Earth observation (EO) is the practice of collecting data on the Earth's biological, physical, and chemical processes using remote sensing technologies and various earth-surveying techniques (European Commission, 2016). In the context of climate tracking, EO-derived data can then be used to develop air pollution and various climate indicators, including wildfires, dust storms, urban green space, and urban particulate matter mortality (Anenberg et al., 2020). While earth observation has traditionally relied on government-funded EO programs that have established satellites like the U.S. and European-government funded Sentinel and Landsat programs or commercial IKONOS, emerging technologies like Unmanned Aerial Vehicles (UAVs) and miniature satellites (e.g. CubeSats) are pushing the boundaries of remote sensing and what it means to collect “big EO data” or “big environmental data” (Sudmanns et al., 2018). Open access policies to EO data through the Sentinel and Landsat programs have also been instrumental in generating huge amounts of data: Sentinel 1, 2, and 3 combined generate in the order of 20 TB of data a day (Esch et al., 2018). So far more than 12 million Landsat images have been distributed across 186 countries due to open data policies that have made rich archives of EO data freely and publicly available (Giuliani et al., 2017). Below we discuss the potential for EO data to address the gaps identified in the previous section regarding primarily self-reported emissions inventories used to assess progress toward climate mitigation.

#### Greater Standardization of Data Management

Remotely sensed EO measurements using satellites and UAVs potentially provide a more standardized method of data collection, providing more consistency and comparability. International organizations like the Committee on Earth Observation Satellites (CEOS), the Global Climate Observing System (GCOS), and the Group on Earth Observations (GEO) have been established to facilitate the interoperability of disparate EO systems. The Quality Assurance Framework for Earth Observation (QA4EO), endorsed by CEOS, provides guiding principles and indicators for ensuring data quality, while the GEO Strategic Plan 2016–2025 promotes the adoption of common standards and disseminates best practices for data management (Sudmanns et al., 2019). These internationally shared standards help to improve the design of algorithms and workflows in data processing.

#### Higher Spatiotemporal Resolution of Data

Regular collection of earth observation data only came online in the 1970s when the United States launched the Landsat sensor capable of measuring four spectral bands at 30-meter spatial resolution. Since then, the range of geostationary and orbital satellites has expanded to provide regular, consistent monitoring of earth phenomena ranging from land cover, oceans, and atmosphere and is why they are considered a possible alternative data source for measuring climate mitigation and adaptation. Sensor technology has advanced dramatically to include multispectral and hyperspectral imaging as well as radar and lidar sensing for the observation of land-use, hydrological, and atmospheric changes (Guo et al., 2015; McCabe et al., 2017). Only two satellites, however, exist for monitoring greenhouse gas emissions from space (Tollefson, 2016), including Japan's GOSAT sensor [National Institute for Environmental Studies (NIES), 2019], which has been operational since 2009 and is capable of measuring column CO_2_ and CH_4_ concentrations, and NASA's Orbiting Carbon Observatory (OCO-2) satellite, which launched in 2014 and provides higher spatial resolution than GOSAT (Monastersky, 2014). Although these satellites were developed for scientific atmospheric observation research rather than for compliance or policy measurement purposes, they have laid critical groundwork for future development of greenhouse gas monitoring through satellite observation that provides regular, consistent and repeated measurements of a range of greenhouse gases from space (Matsunaga and Maksyutov, 2018). Governments ranging from Europe, China, United States, Japan as well as others are already planning a tripling in the number of satellites monitoring just CO_2_ and CH_4_ by 2030 (Tollefson, 2016).

When combined with ground-based emissions estimates, advances in EO have the potential to generate atmospheric, observationally-consistent data that could help improve the accuracy of emissions data and provide verification support (Oda et al., 2019). EO data would complement existing surface-based network atmospheric observations at the regional level, offering higher measurement precision and spatiotemporal resolution that could fill gaps in regions that currently lack surface greenhouse gas or atmospheric monitoring networks, particularly over cities or point sources such as power plants (Duren and Miller, 2012; Ciais et al., 2015). These near-earth and space-based approaches to emissions inventory development use atmospheric transport models to relate surface fluxes of CO_2_ to atmospheric CO_2_ concentrations at a given location in time (Ciais et al., 2015). Such multi-resolution modeling approaches are generally considered labor-intensive and only available for a select number of cities (e.g., Indianapolis, Los Angeles, and Paris) (Davis et al., 2017; Oda et al., 2019), with a need to better assess and quantify associated uncertainty. The Open-source Data Inventory for Anthropogenic CO_2_ (ODIAC) is an example of a 1 x 1 km spatially-explicit fossil-fuel CO_2_ emissions inventory that incorporates point-source CO_2_ emissions data along with satellite nighttime lights data (Oda and Maksyutov, 2011).

Land-based applications (e.g., monitoring of forests, land-cover) are more established in EO, than for atmospheric and climate applications, in the academic literature (Patenaude et al., 2005; Plummer et al., 2006; Goetz and Dubayah, 2011). Remote sensing can better map vegetation types and disturbances in forest cover with “wall-to-wall” coverage, especially in areas where it is not feasible or cost effective to conduct ground-based surveys (Goetz and Dubayah, 2011; Taylor et al., 2020). For example, machine learning techniques have been applied to historical remote sensing datasets of forests and deforestation with high-resolution data from recent years, leading to significant improvements in the proxies for the calculation of above-ground and below-ground biomass, as well as in the identification of correlational signals of forest conservation with concepts of “additionality” (i.e., additional carbon sequestration) and “leakage” (i.e., emissions that result from sequestration efforts) (Meyers, 1999; Baker et al., 2010; Cowie et al., 2012). Such land-use based emissions are frequently contributors to uncertainty in emissions inventories, and the use of EO data has been discussed in policy communities for helping primarily to provide spatiotemporal data with sufficient resolution and enhanced accuracy.

#### Lowering Cost and Proliferation of Sensing Technologies

Advances in sensor and satellite technology, driven largely by start-up entrepreneurs and private sector companies, have introduced low-cost, small-scale satellites that have the potential for lowering the cost of EO for climate monitoring. There are now more working satellites deployed by fledgling space startups than by established government space agencies (McCabe et al., 2017). Private companies have stated plans to lower the costs of CubeSAT satellites, miniaturized space satellites, from $50,000 to $200,000 to even < $10,000 per satellite (Nervold et al., 2016), with anticipated costs even lower for ChipSATs, smaller, postage-stamp versions of CubeSATs (Abate, 2019). The low cost of satellites, UAVs, and other sensing technologies may allow for greater opportunities for data collection, especially in gap filling where other satellites may miss out on daily or more detailed measurements (Witze, 2018). Small and inexpensive drones, for example, can be used for community-based forest monitoring to enable more effective forest management and conservation, which would be useful for tracking climate change adaptation progress (Paneque-Galvez et al., 2014). This trend of lowering costs means more global coverage of the earth's physical, chemical, and biological state than ever before, potentially addressing the problem of incomplete climate data, particularly for land-cover and land-use-based tracking in deforestation, agriculture, and urbanization—key drivers of climate change.

#### Greater Transparency

With EO technology, there is a reduced reliance on climate actors to submit their own data, since a global network of remote sensors could collect climate emissions data instead. When considering emissions related to deforestation, for example, satellites and UAVs can capture multispectral images and laser scanning data of land use changes to determine if forest clearing is within concession areas, allowing for improved transparency and accounting efforts. For example, Page et al. (2002) estimated a loss of 0.19–0.23 gigatonnes of carbon after a 1997 El Niño event in Indonesia using satellite images of a 2.5 million hectare study area in Central Kalimantan, Borneo. The Global Fire Emissions Database (https://www.globalfiredata.org/) compiles satellite EO data on fire activity and vegetation productivity and climate models to estimate monthly burned areas and fire emissions on a gridded scale in near-real time, demonstrating the possibility for EO data to provide timely, transparent and consistent emissions data without requiring actors to self-report data, which may be less accurate and experience time lags.

#### Lowering of Technical Barriers for Emissions Tracking

EO technologies could directly measure greenhouse gas emissions of important polluting sites, thereby reducing the technical barriers to collecting climate emissions data. As mentioned in the previous section, measuring non-CO_2_ emissions such as methane (CH_4_) and nitrous oxide (N_2_O) are more technically challenging for actors to measure than CO_2_ and represent significant sources of uncertainty in emissions inventories. Available ground-based measurements of these pollutants are sparse and limited in their global representation (Frankenberg et al., 2005). Existing CH_4_ monitoring networks are considered inadequate to explain observed trends and variation in atmospheric CH_4_ (Riris et al., 2019). Advances in EO technology have the potential to enhance measurement accuracy for these gases in particular, which primarily result from natural (e.g., wetlands, ruminant animals, rice cultivation) and anthropogenic sources (e.g., fossil fuels; Frankenberg et al., 2005). While a handful of satellites have existed since the mid-1990s for remotely measuring CH_4_ from space, they have primarily been used for detecting hotspots or evaluating emission trends rather than for use in developing emissions inventories, which requires a greater sensitivity to constrain to a more local level, although EO-derived CH_4_ measurements have so far been evaluated to be fairly accurate and show promise for climate policy applications (Jacob et al., 2016; Riris et al., 2019). Governments are planning to deepen EO capabilities for GHG monitoring, including NASA, which is designing the Geostationary Carbon Observatory (GeoCARB) system that will provide as many as 10 million daily observations to measure methane plumes near the earth's surface (Fialka, 2018).

Private sector companies and non-profit organizations are also developing a range of high-resolution satellites to specifically target greenhouse gas monitoring. Google has backed a collaboration between non-profit organizations WattTime, Carbon Tracker, and the World Resources Institute to operate a global network of satellites to monitor the greenhouse gas emissions of large power plants (Arnone, 2019). Private companies like Planet Labs have also started to develop high-resolution earth observation data (with pixel resampling capabilities of 5, 3, or 0.72 meters) that could vastly increase the ability to assess detailed on-the-ground climate data (Planet Labs, 2015). The Environmental Defense Fund is also constructing MethaneSAT, a new satellite that will measure methane pollution from oil and gas facilities (Davis, 2019). GHGSat is another company that is providing high-resolution remote sensing solutions for the tracking of industrial site emissions all over the world. GHGSat satellites and aircrafts can detect light absorbed in a variety of spectral bands to measure concentrations of greenhouse gases including carbon dioxide, methane, and nitrogen oxide, the resolution of which can go down to single meters. Complementing the range of planned government-backed EO satellites, these substantial investments in high-resolution, localized GHG monitoring via EO satellites should bolster global measurement of non-CH_2_ gases substantially.

### Ground-Based Internet-of-Things (IoT)

Complementing the space-based and near-earth sensing capabilities of EO is ground-based IoT. IoT is a network infrastructure in which objects equipped with computing capabilities can communicate directly with each other as well as collect and transmit data to central servers (ITU, 2012; Tzounis et al., 2017). These objects are identifiable through Radio-Frequency Identification (RFID) tags, infrared technology, or unique barcodes (Khan et al., 2012). The number of devices connected to the internet is increasing yearly, and it is predicted that by 2021, 25 billion IoT-related devices will be in use worldwide (Gartner, 2018). Advances in sensor technology, particularly when networked through an IoT infrastructure, could provide more granular data to understand climate change mitigation and impacts. By 2021, the amount of data generated by IoT applications will reach 847 ZB (1 ZB = 10^21^bytes) (Cisco, 2016).

**Figure 1:**
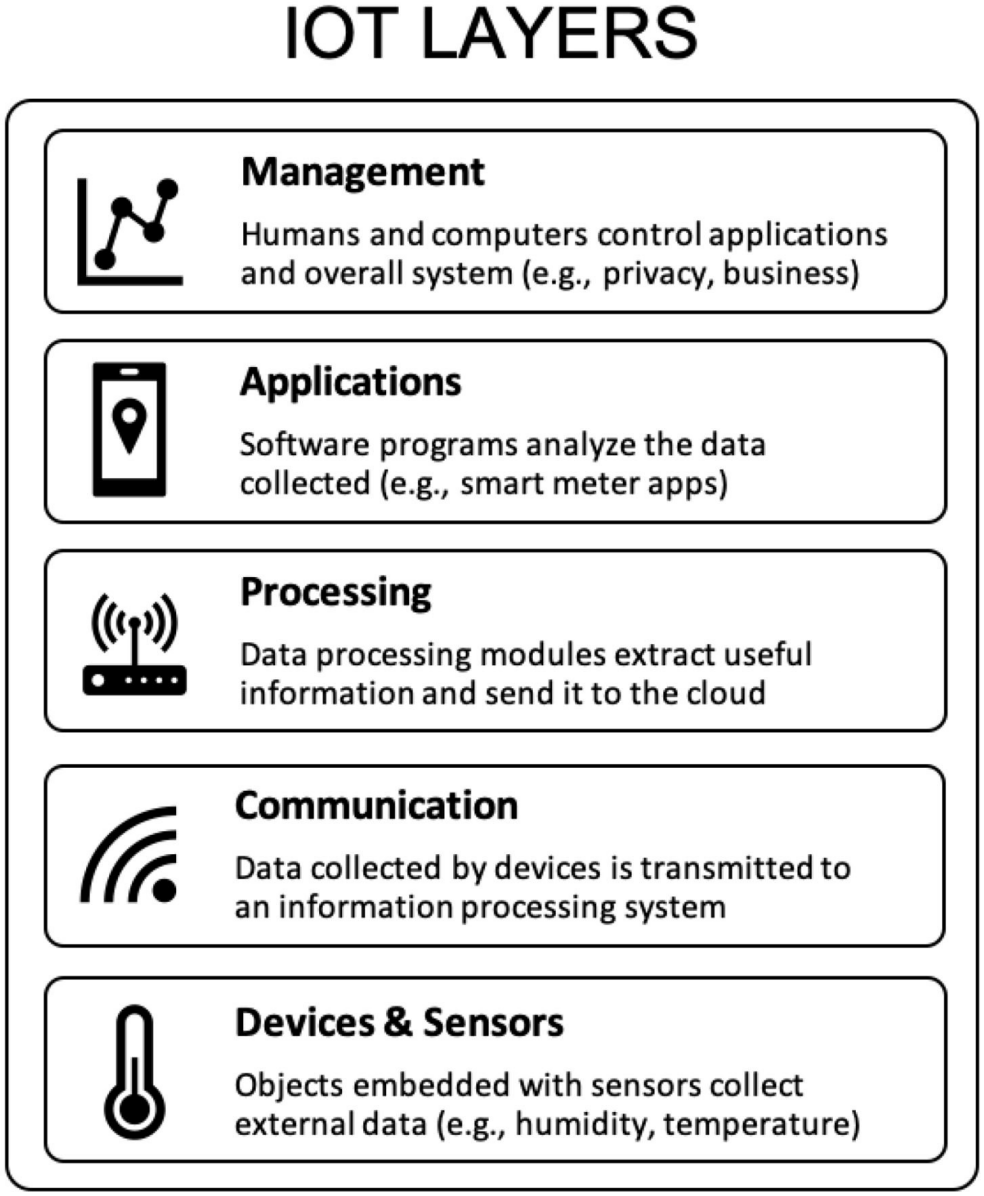
The five main layers of IoT comprise devices, communications protocols, processing systems, applications, and management. Source: authors.

The architecture of IoT can be illustrated through five layers (Figure 1), with the first layer consisting of devices (i.e., physical objects and sensors). Smart meters measuring real-time building energy consumption, soil moisture detectors at crop sites, and even underwater devices measuring water temperature and pressure are a few examples of IoT devices that can exist in a network and provide detailed, real-time environmental data applicable for monitoring progress toward climate mitigation as well as adaptation. The second layer of IoT technology consists of data-transmission technology that sends information collected by a specific device securely to an IoT gateway or information processing system. Devices within the same service can also connect and share data with one another—a critical function that allows for receiving information from networks of devices that can then be managed and stored in a database. Depending on the sensor used within a specific device, the means of transmission may be through 3G, UMTS, Wifi, Bluetooth, or other forms of networking technologies. A processing layer then performs computations to extract useful data before relaying it to the cloud. Crucial to this layer is its feature of automated decision-making, which is based on programmed data processing modules. The application layer can be developed out of the information collected and transmitted from previous layers. The data can then be utilized to better manage various systems ranging from marine ecosystem monitoring to transportation control. The final layer of IoT technology is the management layer, which controls the whole system and can develop varying operational models based on the data received from preceding layers. Together, these layers make up a seamless architecture of ground-based networks and objects that is well-suited for collecting climate mitigation and adaptation data, complementing the efforts of space-based and near-earth remote sensors.

#### Greater Spatiotemporal Resolution of Data

IoT devices can offer high-resolution temporal and spatial data for climate mitigation and adaptation monitoring. For example, in Norfolk, Virginia, where flooding events are an increasing occurrence, ultrasonic sensors can detect minute changes in the distance between water level and ground to provide real-time flood analysis (Carlson et al., 2019). In the electricity sector, networks and sensors can be easily implemented to collect energy readings *in situ* from PV or wind power systems, sending that data to remote IoT gateways for preprocessing, storage, and transmission to the cloud for monitoring and analysis. Open IoT platforms such as Raspberry Pi and LoRa can further enhance efficiency in renewable energy monitoring systems. Specifically, a low-cost, wide-area LoRA network using <1 Ghz frequency does not require a telecommunications base system and can allow for consistent data collection using end-to-end LoRa modems to send energy readings to microcomputers like Raspberry Pi (Choi et al., 2018).

#### More Complete Data

The low cost and ease of deployment of IoT devices and networks significantly reduce the barriers of data collection. For example, the rise of cost-effective water quality sensing nodes and probes using commercially available off-the-shelf parts could pave the way for large-scale implementation and robust environmental data collection and monitoring for assessing urban water quality targets in national and subnational adaptation plans (Postolache et al., 2014). In buildings and smart homes, IoT devices could be used for efficient and inexpensive appliance load monitoring—a single smart meter equipped with learning and detection processing is capable of collecting electricity consumption data of multiple appliances in a building or home for energy efficiency target tracking (Ma et al., 2018). IoT could also be used for energy tracking and optimization in the manufacturing sector. Wang et al. (2018) introduce a real-time energy efficiency optimisation method (REEOM) which utilizes RFIDs and smart meters to monitor the flow of materials as well as the consumption patterns of machines. The IoT devices then send information to a central server using Wi-Fi, RS 485, or Zigbee for analysis. These energy monitoring systems allow for energy-aware process scheduling and reduced resources wastage, and are also able to continuously collect data without network or supply chain interruption.

Other notable systems for energy monitoring include the OpenEnergy Monitor and ACme. OpenEnergy uses open source Arduino boards in a three-phase power metering system that measures apparent and real power, and Root Mean Square (RMS) voltage and current, while ACme measures active, reactive, and apparent power using the wireless technology 6LoWPAN for small devices with limited processing (Jiang et al., 2009; Pease et al., 2018). These different monitoring systems could help to push the boundaries of indirect emissions accounting in a multitude of sectors. In smart city initiatives, connected wearables, sensors, and actuators can help improve urban governance, environmental monitoring, and sustainable living. The IoT European Large-Scale Pilots (LSP) Programme was launched in 2016 to test the scalability of IoT applications and the interoperability of EU-based IoT platforms, such as Open-IoT and FIWARE (Meiling et al., 2018). The programme includes pilot projects which have been deployed in various European cities and focus on using end-to-end sensor applications for collecting measurements on issues of aging, food security, and autonomous vehicles.

#### Greater Transparency

The proliferation of open source electronic prototyping kits such as Arduino, Raspberry Pi and.Net Gadgeteer has given rise to a new wave of citizen science, increasing data transparency and reducing the reliance on major climate actors to share and report data (Salim and Haque, 2015; Fritz et al., 2019; Hsu et al., 2020b). Users can share code through online communities and use these microelectronic kits to build their own sensors and actuators to measure a range of ambient environmental conditions, including temperature, humidity, and barometric pressure. Sensor.Community is an example of an open-source platform in which civic-minded contributors from all over the world build their own IoT devices and join a global network, taking part in various campaigns and community projects that generate new environmental data (e.g., PM_2.5_, PM_10_, noise, relative humidity; Sensor.Community, 2020). Similarly, the Air Quality Egg is a WiFi-enabled device that uses sensors to measure changes in the levels of NO_2_, CO_2_, CO, O_3_, SO_2_, particulates, and volatile organic compounds (VOCs). Citizens upload measurements to the cloud and have access to a global network of air quality data (Air Quality Egg, 2014).

The integration of IoT into processes of inventory management can also introduce more transparency between actors in supply chains, bringing about more efficient planning and sustainable production. Specifically, in the agricultural sector, where supply is especially unpredictable, Near Field Communication (NFC) tags may be used to tag produce, and Android devices with NFC readers and GPS antennae could identify and track information (e.g., location, origin, certification, price) on that produce through the internet (Srinivasan et al., 2017). Such a system could prevent wastage and waiting times as well as optimize supply chain integration, reducing overall emissions within the agricultural sector.

#### Lowering Technical Barriers for Emissions Tracking

IoT technology is capable of obtaining technically challenging data in both adaptation and mitigation domains. Low-power and long-range IoT devices can be installed at various industrial sites to capture GHG emissions in real time or near-real time. An example includes Envira IoT's Nanoenvi devices, which feature multiple probes and sensors and can be installed in industrial plants to monitor the concentrations of process gases. They can withstand harsh conditions and can be integrated with various IoT control centers using communication protocols (e.g., message formats, communication and transport networks, M2M networks). In smart city initiatives, open-source, long-range, low latency, and low power wireless network standards like DASH7 may be integrated with IoT devices to collect information on temperature, light, humidity, and pressure for the monitoring of green infrastructure (Le et al., 2019). These devices could also be employed in the adaptation domain, where sensors measuring water level, water speed, temperature, humidity, and turbidity, can generate warnings for flood detection systems. In the construction sector, specifically, an IoT-based system could make use of a distributed sensor network (e.g., RFID) to collect real-time emissions data to improve carbon emissions monitoring at different stages of the construction process (Mao et al., 2018).

## Current Limitations of EO and IoT Data Collection Solutions

Even with the advent of EO and IoT technology, the full potential for data collection has yet to be realized due to a range of challenges. This section examines these remaining challenges according to our defined categories from the section Defining the Problem Space: Issues With Data Collection and summarizes them in Table 1. A common theme in the literature regarding the generation of big earth data is the lack of existing methods, infrastructures, and interaction modes to handle the scale and domain peculiarities related to these data types. An in-depth discussion of the storage and processing requirements needed to process these large-scale data extends beyond the scope of our review here, but it is nonetheless worth highlighting some of the general challenges inherent to the task of integrating the large stores of EO and IoT data that currently exist or will be generated. Sudmanns et al. (2019) identify challenges attached to big Earth data along three dimensions: technological, methodological and societal. In the societal domain, the most obvious challenge is that EO or IoT data generation does not automatically translate into actionable insight or usable knowledge. Certain human-centered processes, such as human interactions with natural systems, for example, may require additional parameterization in combination with large-scale EO and IoT data to make sense of signals in the data. As climate change is a complex process operating at the nexus of geophysical, biological, and social systems, these complexities fundamentally make it difficult to assess, even with limitless data.

Leveraging EO and IoT data collection for climate change mitigation will further require technological innovations to store, process, interact and analyze data. “Big Earth data” or “big environmental data” is often characterized by non-repeatability, uncertainty, multi-dimensionality, and computational complexity that will require radical new modes for how users interact and produce information from these new data (Sudmanns et al., 2019). According to Giuliani et al. (2017), these dimensions will require new storage, processing and retrieval approaches to leverage the full “information power” of this new data to broaden its uptake amongst a range of users and support decision-makers with knowledge required. Interoperability between EO and IoT systems, data types and standards, which currently do not exist (Sudmanns et al., 2019), will further be required to effectively utilize and apply the growing pool of EO and IoT data to assess climate change outcomes. Ogle et al. (2013) argue for advanced software systems that include relational databases and graphic user interfaces that allow users to easily input and extract data without knowledge of computer code, pointing to the example of the Agriculture and Land Use National Greenhouse Gas Inventory (ALU) Software (www.nrel.colostate.edu/projects/ALUsoftware/).

To organize, store and facilitate analysis of large EO data generation, the idea of a “data cube” has been introduced that is equipped to handle the spatial and temporal multi-dimensionality as well as the sheer size of EO and IoT data using new infrastructures, software implementations and user interfaces (Giuliani et al., 2017; Baumann, 2018; Sudmanns et al., 2019). Examples include EarthServer, the Swiss Data Cube (SDC) (Giuliani et al., 2017), and Digital Earth Australia, formerly known as the Australian Geoscience Data Cube, (AGDC) and represent a promising emerging solution for storing and analyzing large-scale Earth-related data in an efficient way (Sudmanns et al., 2019). Sudmanns et al. (2018) refer to the data cube concept as “the backbone of modern big Earth data analytics” and represent great promise for lowering barriers for these data to be applied, particularly in combination with “soft” technological infrastructure solutions such as Google Earth Engine (Gorelick et al., 2017), which applies Google's distributed cloud computing technology to enable easy access and analysis of entire archives of satellite EO imagery, and ML techniques that are increasingly being applied to IoT generated data.

### EO Limitations

#### Lack of Technical Capacity to Interact With Collected Data

While deriving GHG emissions data from EO represents a promising solution to existing information gaps, translating raw EO data into actionable insight for the climate policy community is not a straightforward task. Existing EO sensors that currently monitor GHG emissions from space were not designed for tracking policy or treaty compliance (Witze, 2018), and EO data that do exist for GHG monitoring still need to be validated and shown to be more accurate than ground measurements (Tollefson, 2016). There are efforts underway to develop Analysis Ready Data (ARD) data products from EO to lower the barrier for users to utilize the data directly, incorporating pre-processing and calibration steps that currently prevent much of EO data to be readily applied by non-expert users (Giuliani et al., 2017). EO data currently remain underutilized for several reasons, including the fact that existing stores of data have outpaced our ability to analyze them; a lack of technical know-how and infrastructure to appropriately access and process them (Giuliani et al., 2017).

#### Attribution and Research Uncertainties

There are also key scientific challenges related to EO data and its relevant technology that must be determined before it can be immediately applied in the climate policy domain. Despite the ability of EO to collect repeated atmospheric observations on the increase of CO_2_ concentrations, their attribution to actions of any actor, whether local or national government or corporate, has not been possible (Duren and Miller, 2012). This challenge is due to the fact that the existing generation of EO satellites measure CO_2_ within a column of air and do not pinpoint where the emissions are actually derived—the critical question for compliance and policymakers (Tollefson, 2016). Until these key scientific hurdles, including uncertainties in the amount of CO_2_ the land and oceans uptake, can be resolved, it will remain challenging to use EO data to directly assess the impact of mitigation policies or to predict global warming. In terms of monitoring carbon losses from deforestation, there are also no standardized practices for measuring forest biomass, a key component of forest carbon stock assessment, through remote sensing at regional and national scales (DeFries et al., 2007; Tollefson, 2016; Witze, 2018).

#### Spatial Resolution and Intermittency

While the spatiotemporal resolution of EO satellites and sensors is improving by the day, the use of EO data still faces real trade-offs between temporal frequency, spatial coverage and image resolution. For example, while remote-sensing based systems have been used for fire detection, monitoring, and management, EO sensors' moderate range and poor spatial resolution make them inadequate for forecasting fire danger (Chowdhury and Hassan, 2015). For example, while the aforementioned ODIAC fossil-fuel CO_2_ emissions inventory is generally in agreement with other GHG inventories, uncertainty increases when the data is applied to evaluate spatial extents smaller than 1 km × 1 km (Oda and Maksyutov, 2011; Jonas et al., 2019). In the case of vegetation phenology, the study of seasonal plant life cycle, which is useful for anticipating climate impact and adapting to climate change, satellite imagery is accurate in estimating plant behavior in deciduous forests but less so in tropical forests (Hmimina et al., 2013). There are also issues of intermittency in imaging: only two to five percent of data GOSAT collects can be used for CO_2_ and CH_4_ calculation, due to requirement of clear-sky conditions, which is a major limitation for EO data in serving as an alternative data source for GHG emissions monitoring [National Institute for Environmental Studies (NIES), 2019].

#### Inventory Discrepancies

There is also a lack of agreement between inventories developed from satellite remote sensing data sources and self-reported emissions inventories (Weiss and Prinn, 2011), which requires clear communication of uncertainties involved when applying digital technology-based data collection methods because this awareness can help facilitate decision making (Jonas et al., 2019). Some limitations of remote sensing can be overcome by combining different data technologies. For example, remote sensing data can be triangulated with data from airborne photography to model temperate glacier flow at a much higher resolution (Trouve et al., 2007). The World Meteorological Organization (WMO) has established the Global Greenhouse Gas Information System (IG3IS) initiative, which is comprised of scientists and stakeholders from around the world to develop methodologies for combining atmospheric GHG concentration measurements, such as those derived from EOs with spatially and temporally explicit emissions inventory data from the ground which could include data collected through IoT (Decola and Tarasova, 2017).

### IoT Limitations

#### Privacy and Security

As with all digital technology collecting real-time information of individual actors, privacy and security is critical. The data collected by IoT-networked devices often contain personally identifiable information or proprietary information and are continually at risk of being exposed or stolen. Yet the lightweight and low-power nature of IoT devices—a feature that makes these “Things” such efficient and scalable data-collectors in the first place—precludes them from using security mechanisms with high energy and computation overheads like antivirus or intrusion detection systems (Zhang et al., 2014; Dorri et al., 2017). The lack of readily available security protocols and data anonymization schemes for IoT devices may prevent their scaling and applicability.

#### Identification and Authentication

Another key issue with respect to IoT-derived data is object identification. At present, there are billions of unique IoT device identities that exist. The current method of device identification, the Domain Name System (DNS), is a naming infrastructure that is not immune to DNS spoofing, DDoS attacks, and man-in-the-middle attacks, which corrupt the integrity of records (Zhang et al., 2014). As an increasing volume of IoT devices are produced and connected, developing a dynamic and efficient system that can assign and manage the unique identities of each device is essential.

#### Heterogeneous Devices and Protocols

Additionally, devices produced by differing manufacturers may not utilize the same technologies and services, rendering the interoperability of various devices and sensors uncertain. A means of standardization is therefore necessary to ensure that devices are still compatible with one another. In reality, there are more than 300 current IoT platforms in the market, including platforms developed by major corporations such as Amazon, Cisco, IBM, Apple, Google, Microsoft, and Qualcomm (Noura et al., 2019). Differences in each platform's IoT infrastructure, standards, proprietary protocols, and formats create closed ecosystems wherein the IoT technology and services between platforms are incompatible with one another (Noura et al., 2019). The aforementioned problem of data heterogeneity in climate data is therefore not resolved with IoT. The lack of interoperability and standardization jeopardizes security as poorly created programs can easily slip through the cracks and onto the market. These software vulnerabilities can then lead to malware or backdoor problems, in which malicious actors use reverse engineering techniques to control the device (Zhang et al., 2014).

Recently, numerous institutions have attempted to address the interoperability of IoT technology through proposed policies and/or other means of standardization. The European Commission has been involved in the research and development of a number of projects related to the IoT, and in 2015 adopted the “Digital Single Market Strategy.” This strategy highlighted the necessity of avoiding the fragmentation of device technologies and services. Following the adoption of this strategy, the European Commission has promoted and enacted a number of studies and policies that address the development of a single market. These projects include a 2017 “European data economy” initiative that proposed policy dealing with the flow of data across EU country borders, a 2019 “Cluster Study” which has surveyed the current enterprises, research organizations and academics that are involved in the innovation, development and deployment of IoT technologies and applications, and IoT research and innovation objectives in the European Commission's “Horizon 2020” program (European Commission, 2019). Further, processing methods of heterogeneous data are advancing, with new fusion techniques and semantic approaches that can merge structured, semistructured, and unstructured information sources into a unified data layer. These tools include Natural Language Processing (NLP), entity recognition and linking (e.g., DBpedia Spotlight), data lakes, and data virtualization (Wang, 2017), allowing IoT and EO data to be integrated with each other as well as with information from more traditional streams (e.g., government agencies, national statistical offices; Fritz et al., 2019).

## Discussion: Toward the Next Generation of Tracking Climate Change

Our review shows that digital data technologies such as EO and IoT have the potential to address major gaps in existing climate data, particularly for the purposes of tracking and assessing progress toward greenhouse gas mitigation. In this section, we outline critical considerations for a digital, next-generation solution based on digital ledger technologies (DLTs) to integrate and apply EO and IoT technologies for climate change monitoring and tracking. We discuss the potential for DLTs to provide a more transparent and secure infrastructure for housing multiple data streams, introduce privacy techniques to encourage actors to report data, and build in automatic governance mechanisms that would help track progress toward external frameworks like the Paris Agreement. We also identify critical areas of research that are needed for such a DLT-based system to become realized, including energy consumption, storage costs, and data quality.

DLTs such as blockchain have been discussed in a range of applications outside of its origin in the cryptocurrency domain, including emissions trading schemes (ETS; Khaqqi et al., 2018), precision or ICT-enabled agriculture (Lin et al., 2017), and renewable energy incentives for electric vehicles (Zhang et al., 2018), among others. While digital ledgers are record-keeping databases that provide a common chronological order of transactions within an organization, distributed ledgers enable multiple actors to each hold a copy and constantly update it. In a DLT system, there is no central server or central point of failure for data to be tampered. The result is an immutable chain of “artifacts and claims that can be traced back to the original raw data” (Sicilia and Visvizi, 2019), allowing for a user to understand the origins of the data, any processing algorithms, or claims made—important considerations for the purposes of establishing credibility in government and policy contexts where legacy IT-enabled data management systems typically used are prone to cyber-attack, inaccurate data, distortion and manipulation (Lin et al., 2017).

**Figure 2:**
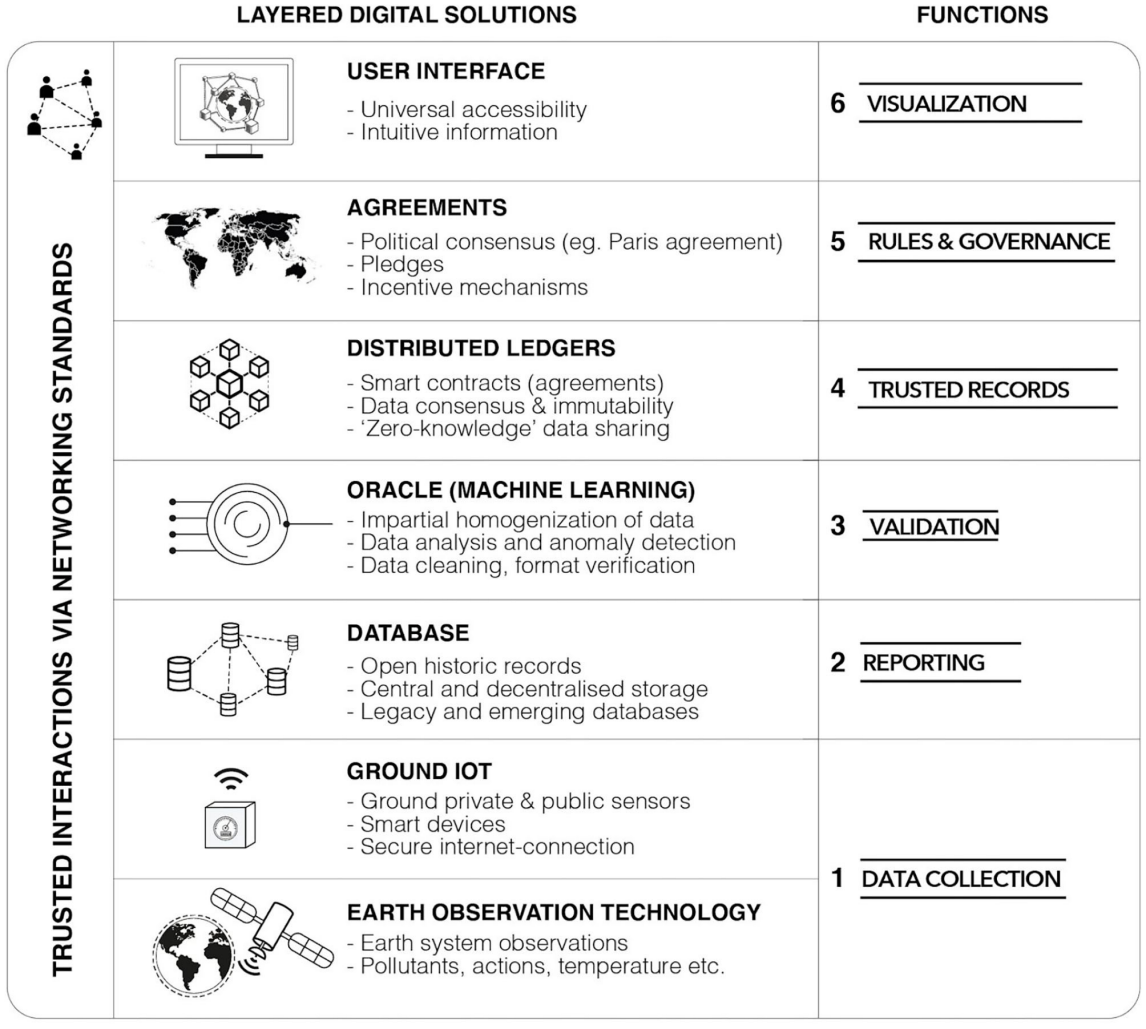
Overview of different digital data collection technologies and layered data solutions within the climate sector. Source: authors.

In Figure 2, we illustrate how digital data collection technologies discussed in this review paper could form the foundation of a “next generation” approach to climate data tracking based on DLTs. First, IoT and EO technologies collect data (layer 1), the latter operating alongside existing ground-truth sites that act as the calibration and triangulation for ensuring data quality. The data could then leverage peer-to-peer distributed storage systems, such as the InterPlanetary File System (IPFS) and Storj, to facilitate tamper-resistant data pre-processing, sharing, and storage (layer 2). Collected, processed data could then enter a soft-infrastructure component to train and use machine learning models for further evaluation and analysis of incomplete datasets (layer 3). Once data is validated by machine learning or external data feeds (i.e., oracles), immutable, time-stamped data can be recorded on a DLT ecosystem, and smart contracts, which are computer programs or transaction protocols that execute agreements between parties (Christidis and Devetsikiotis, 2016), could communicate that certain benchmarks or targets have been met (layer 4), paving the way for agreed-upon incentives to be paid out, or for human, organizational, and computer actors to formulate further agreements and governance mechanisms based on established goals (e.g., Paris Agreement) (layer 5). A user interaction layer would facilitate the use of this multi-layered digital ecosystem, drawing upon the experience of platforms and interfaces currently being developed in the EO and IoT communities to translate raw data streams into usable and actionable information (layer 6).

### Key Advantages of a DLT-Based Climate Measurement and Tracking System

#### Data Privacy

DLT systems can make use of privacy technologies, such as zero-knowledge proofs and homomorphic encryption, to address some of the privacy concerns associated with data sharing by protecting sensitive and proprietary data (Kosba et al., 2016). Zero-knowledge proofs allow data to be verified without parties having to know what that data actually is, and are used by DLTs such as Ethereum and Zcash, while homomorphic encryption enables computations on encrypted data (Homomorphic Encryption Standard, 2017; Koens et al., 2018). Applied to climate data, zero-knowledge proofs could allow data-reporting actors and objects to prove that they belong to a system or consortium and interact with it without necessarily revealing their identities. This identity protection could be advantageous to incentivize actors concerned that revealing certain climate data would reveal trade secrets (i.e., through production efficiency metrics). Similarly, homomorphic encryption presents the opportunity for statistical checks to be performed on encrypted granular emissions data without exposing the actual data (Hsu et al., 2020a).

#### More Efficient Governance

DLTs can also provide rules for decentralized governance and consensus, utilizing smart contracts to execute transactions, data transmission, and voting more efficiently and transparently between actors (Catalini and Gans, 2016; Underwood, 2016). These elements could allow for a greater inclusion of climate stakeholders and lead to a more democratic network for knowledge-sharing and decision-making. Decentralized autonomous organizations (DAOs), for example, are entities powered entirely by smart contracts. The rules of the organization are encoded and enforced in a DLT-system, where members democratically make decisions, control funds, and manage information without the need for financial or legal intermediaries (Norta, 2015). In the context of a global integrated climate accounting platform, DAOs could be useful for creating efficiently automated data sharing communities, where reporting organizations agree to transparent rules on how data should be collected and submitted. For example, the Paris Agreement and Rulebook provides a set of formalized protocols for how countries should develop their emission reduction contributions and communicate and report progress toward them. DAOs enforce accountability by enshrining these rules in code and executing them automatically through smart contracts.

### DLT Limitations

Several considerations would need to be addressed for such a next-generation climate data and tracking system to be realized. These challenges are inherent to the digital solution technologies proposed in Figure 2 and not necessarily specific to climate change, which means such a framework, and the challenges identified, could potentially be applied to address other domains as well.

#### Energy Consumption of Consensus Protocols

To maintain and constantly update the state of a distributed ledger, participants within a system need to adhere to a consensus mechanism, which provides the necessary rules and fault-tolerance for validating transactions (Mahmood and Wahab, 2018). In the case of a DLT-based climate data system, a consensus protocol for exchanging and recording data would need to address the common criticism of high energy consumption, as seen in the proof-of-work approach (used by Bitcoin, for example), which relies on intensive computer calculations. There are, however, other methods of validating transactions that are significantly more efficient in terms of energy usage. These include the proof-of-authority and proof-of-stake algorithms, which rely on actors' identities or contributions of funds, respectively, instead of computational power. Proof-of-authority's strict identity requirements and limited number of validators, however, could decrease participation within a network, thereby reducing its decentralization. Similarly, proof-of-stake could lead to a centralization of resources where wealthy validators enjoy economies of scale and crowd out nodes with smaller stakes. Full decentralization of climate data, however, is not the main goal of our proposed integrated system. A detailed discussion of the tradeoffs within the existing and growing universe of consensus protocols is beyond the scope of this paper. Deciding the rules for data submissions and recording in a way that is not energy-intensive while enhancing trusted interactions, security, privacy and efficiency will be key research tasks for designing a next-generation DLT-based climate data tracking system.

#### Cost of Data Storage

Storing raw data on distributed ledgers can be prohibitively expensive, since every node in a network typically needs to synchronize and keep a copy of all uploaded information. Fortunately, distributed data storage systems, such as IPFS or Storj (Zheng et al., 2018) greatly reduce the storage size necessary for information, hence reducing overhead costs for data storage. A DLT-system, combined with a decentralized storage solution, provides a “disruptive approach” (Sicilia and Visvizi, 2019) for archiving digital resources, which will necessarily demand greater storage space with the introduction of massive EO or IoT-linked DLT systems that we suggest are the future of climate mitigation tracking. For example, in the most well-known application of blockchain, the Bitcoin network, transaction data can take up tens of thousands of bytes in storage while an IPFS record is only a few tens of bytes (Zheng et al., 2018).

#### Garbage in, Garbage Out

A common criticism of DLT is the lack of data quality at the source. While DLTs provide a tamper-evident and tamper-resistant trail of trust, it is often difficult to ensure raw data quality at the moment of input (e.g., EO and IOT data streams), which could lead to a trail of immutable but ultimately inaccurate data. Some of the most significant technology advances in response to this concern have come from the development of decentralized identifiers (DIDs) and their use in verifiable credentials that define the roles of holders, issuers and verifiers across complex networks (Davie et al., 2019). The application of DIDs within a network to establish trusted interactions, as in the case of the “Trust over IP stack” (Davie et al., 2019), can provide the needed rules and governance to ensure the data is trustworthy across the different layers outlined in Figure 2. This requires IoT devices to be registered as specific “agents” within the network, and have pre-programmed disclosure settings, even with privacy tools such as zero-knowledge proofs, irrespective of who owns the IoT and where the data is stored (Huh et al., 2017). In systems with multiple human and organizational actors, Hsu et al. (2020a) have proposed a DLT-based solution in which members belonging to a consortium are economically incentivized to report accurate data. Members back their own claims of accuracy or challenge those of others by staking specific funds on a blockchain, which are then slashed or increased based on an independent audit.

Through this proposed integrated system, we aim to lay the groundwork for further examination of how DLTs could introduce new data collection and knowledge discovery architectures for climate change. Groups such as the World Bank and Climate Ledger Initiative have already developed some of the foundation for examining how DLTs could be used for a range of climate-related use cases in finance, adaptation, and mitigation (Fuessler et al., 2018; World Bank Group, 2018). The UNFCCC has even supported the launch of a new Climate Chain Coalition for the sharing of domain and technology expertise [United Nations Framework Convention on Climate Change (UNFCCC), 2018]. Beyond this initial exploration, we envision technologists, policymakers, and scholars collaborating to build a pilot DLT-based climate tracking system that would address the challenges identified in this discussion, including clarifying roles and incentives for participation and data collection, and operationalizing consortium-specific governance mechanisms toward a more unified climate action ecosystem.

## Conclusion

The rise of data collection technologies has resulted in new opportunities for tracking progress toward global climate change mitigation. Earth observation sensors like satellites and UAVs, together with IoT devices and networks, could offer more transparency, more standardized and complete data, as well as solve important technical challenges of emissions measuring. A future integrated climate monitoring and accounting system that leverages digital data collection technologies has the potential to introduce a new level of large-scale data availability, transparency, and standardization for assessing progress toward climate change mitigation. A digital data ecosystem based on distributed-ledger technology that integrates different data streams from self-reported data and legacy databases to EO/IoT digital data collection technologies could amplify the amount of data available to develop a more complete picture of global climate emissions. To realize such an integrated digital climate data ecosystem, however, many challenges will need to be addressed, some of which relate to EO and IoT technologies themselves, and others that relate more generally to the challenge of processing and analyzing the anticipated massive amounts of data that will be generated as a result of these data. Issues of storage, processing, and new analytical approaches will be required to fully leverage the large-scale data generation of EO and IoT technologies. Emerging technologies such as distributed ledger and file storage systems, such as IPFS, provide further potential to link together EO and IoT data streams with existing databases and to introduce a range of other mechanisms to ensure transparency, privacy, and governance that can support next-generation tracking of climate change mitigation.

## Author Contributions

AH: research design, conceptual framework, research, writing and analysis. WK and MW: research, writing, analysis, and figure design. NG: research, writing and analysis. Authors contributed to the article and approved the submitted version.

## Conflict of Interest

The authors declare that the research was conducted in the absence of any commercial or financial relationships that could be construed as a potential conflict of interest.
